# Everybody's Page

**Published:** 1889-12-07

**Authors:** 


### Everybody's Page.

A freshly caught cold is sometimes greatly relieved by washing the nostrils and gargling the throat with a weak solution of salt.

The origin of ambergris has recently been asserted to be a diseased secretion of the spermaceti whale which is found floating on the surface of the sea, and has a smell which, though exceedingly nauseous when in its pure state, gives mellowness to mixtures.

A danger which, according to Mr. Hitchcock, the American chemist, causes great dread to the Japanese is that of poisoning which results from fresh lacquer obtained from the juice of a tree. The sap, when it first exudes, is a greyish-white viscous fluid, which soons turns yellow, and when it is exposed to the air becomes black.

Some herbalists still have shops, and a well-stocked store will contain between two and three hundred different kinds of barks, herbs, and roots. Those most commonly used are hoarhound, sarsaparilla, catnip, camomile flowers, yellowdock, burdock, sassafras, mandrake, cherrybark, stillingia, and wintergreen.

The "heiress of Heron Court," or more correctly the locally notorious but generally obscure Mrs. Day, whose death took place a short time ago, caused the late Lord Malmesbury much vexatious trouble by her claim to Heron Court. A husband who can say of his wife that "you might turn a tree if you could turn her," is not altogether to be envied.

Mr. Stanley pays a very high tribute of praise, in his letter of August 17, to the devotion, under very trying circumstances, of Dr. Parke to the sick. However attentive other surgeons might have been, it would not be given to many to love cases which would repel most men on account of "the odours emitted, and the painfully qualmish scenes." Mr. Stanley's book, when it appears, promises to be a record of unparalleled heroism amidst unexampled trials.

That useful tuber the potato can, it seems, be turned to new account, so there is an opportunity for Paddy to set up a fresh industry. A composition of the nature of paint, it is asserted, can be made by taking two pounds of peeled potatoes and boiling them in water, mashing them, and then diluting them with a pint and a half of water and passing them through a hair sieve. Then dilute four pounds of Spanish white with about six pints of water, and add to the potatoes. The result is a milk-white colour. The mixture can be made of various colours by using different ochres, etc., and dries so rapidly that two coats may be applied immediately one over the other.

Fish-soup is a dainty better known to our economical neighbours on the Continent than to most English cooks. If a cod's head and shoulders or a piece of turbot be boiled in less water than usual, and with less salt, all that is necessary to convert the remains into stock is to replace in the water the bones, skin, etc., of the fish when it comes from the table, with vegetable garnish, omitting turnip, but adding a blade of mace, and boil for an hour and a half. Not more water should be used than will cause the stock, when strained and cold, to assume the appearance of a clear limpid jelly. This broth is the best of all foundations for bouillabaisse, bisque, oyster soup, or fish mulligatawny. Fish stock is also very good thickened with a liaison of a little cream and two yolks of eggs, or simply strained and served over poached eggs, one in each plate. It makes a capital light dish for an invalid who is tired of beef-tea and similar commonplace sick-room diet.

Never eat cooked and uncooked fruit at the same meal if you would avoid acidity or indigestion—so says Mrs. S. H. Hunter, the advocate of vegetarianism. And the reason why we should never eat cooked and uncooked fruit at the same meal is that they digest unequally.

Catarrh snuff is clearly most efficacious. Gentleman, to village cobbler: "What is that powder you are using?" Cobbler: "It's catarrh snuff." Gentleman: Is it any good?" Cobbler: "Well, I've had catarrh for more'n thirty years an' I've never took nothin' for it but this."

What holds good of fruit in a temperate climate like that of Great Britain does not do so in the tropics, where fruit should only be eaten sparingly in the early morning, and never later in the day. Fruits with hard skins or numerous seeds should always be avoided in hot climates.

Oleite, an American preparation for the skin, is prepared from castor oil by treating it with sulphuric acid at a low temperature. The finished substance is a transparent, jelly-like liquid, with acid taste and slight odour; it is soluble in water, alcohol, chloroform, and essential oils.

The cultivation of the native poppy in China is making enormous strides, and the danger of famine with which the authorities have to grapple in any exceptional year will be largely increased, owing to so much of the land being devoted to the cultivation of the poppy instead of to food staples.

Castor oil chocolate does not sound tempting; but, for the purpose of facilitating the administration of castor oil, its incorporation with finely-powdered cocoa, deprived of its natural oil, and vanilla or any other pleasant flavouring substance, and sugar is suggested. The whole ingredients are ground on a heated slab, and then turned into moulds and allowed to cool.

Few people nowadays have faith in the remedies of herbalists and old country women, yet the trade of these people still lingers. A plant highly esteemed among herbalists as a consumptive remedy is a low-growing one called bugle weed. It is sometimes given in the form of an infusion in order to prevent bleeding at the lungs. Wormwood, when moistened with hot water, mixed with salt, and laid on flannel for a poultice, is said by herbalists to take down swellings much quicker than arnica does.

In these days of high pressure a child's mind is too frequently overburdened by the endeavour to instil into its head an excess of knowledge in a short space of time. An amusing instance of this came under our notice a short time ago. A little girl, on her return from the day school which she attended, was asked by her father what she had been learning. "Oh," she replied, "I've been having an object lesson." "And what," said her parent, "have you learnt?" "Well, I learnt that sponge is an opaque substance which grows on sheep's backs."

It is reassuring to hear from Sir W. Moore that it is improbable that leprosy, as a disease, will ever become common in this country. Yet, if one of the great aids to the increase of leprosy is want of proper food and clothing, we fear that, given other things favourable to the disease, there is fertile soil for the communication of it not far from our own doors. Nervous people will be comforted by the knowledge that leprosy is not contagious by slight contact, but is generally, if not always, communicated or contracted by an abrasion of the skin coming into contact with a leprous discharge.

